# Early-phase ^18^F-Flortaucipir tau-PET as a proxy of brain metabolism in Alzheimer’s disease: a comparison with ^18^F-FDG-PET and early-phase amyloid-PET

**DOI:** 10.1007/s00259-024-07063-4

**Published:** 2025-01-24

**Authors:** Cecilia Boccalini, Debora Elisa Peretti, Gregory Mathoux, Leonardo Iaccarino, Federica Ribaldi, Max Scheffler, Daniela Perani, Giovanni B. Frisoni, Valentina Garibotto

**Affiliations:** 1 Laboratory of Neuroimaging and Innovative Molecular Tracers (NIMTlab), Geneva University Neurocenter and Faculty of Medicine, University of Geneva, Geneva, Switzerland; 2 Division of Nuclear Medicine and Molecular Imaging, Geneva University Hospitals, Geneva, Switzerland; 3 Eli Lilly and Company, Indianapolis, USA; 4 Memory Clinic, Geneva University Hospitals, Geneva, Switzerland; 5 Division of Radiology, Geneva University Hospitals, Geneva, Switzerland; 6 Nuclear Medicine Unit, San Raffaele Hospital, Milan, Italy; 7 CIBM Center for Biomedical Imaging, Geneva, Switzerland

**Keywords:** Neurodegeneration, Early-phase tau-PET, ^18^F-FDG PET, Perfusion, ^18^F-flortaucipir

## Abstract

**Purpose:**

As dual-phase amyloid-PET can evaluate amyloid (A) and neurodegeneration (N) with a single tracer injection, dual-phase tau-PET might be able to provide both tau (T) and N. Our study aims to assess the association of early-phase tau-PET scans and ^18^F-fluorodeoxyglucose (FDG) PET and their comparability in discriminating Alzheimer’s disease (AD) patients and differentiating neurodegenerative patterns.

**Methods:**

58 subjects evaluated at the Geneva Memory Center underwent dual-phase ^18^F-Flortaucipir-PET with early-phase acquisition (eTAU) and ^18^F-FDG-PET within 1 year. A subsample of 36 participants also underwent dual-phase amyloid-PET (eAMY). Standardized uptake value ratios (SUVRs) were calculated to assess the correlation of eTAU and their respective ^18^F-FDG-PET and eAMY scans. Hypometabolism and hypoperfusion maps and their spatial overlap were also evaluated at the individual level visually and semiquantitatively. Receiver operating characteristic analyses were performed to compare the discriminative power of eTAU, FDG, and eAMY SUVR between A-/T- and A+/T + participants.

**Results:**

Strong positive correlations were found between eTAU and FDG SUVRs (*r* = 0.84, *p* < 0.001) and eTAU and eAMY SUVRs (*r* > 0.87, *p* < 0.001). Clusters of significant hypoperfusion with good correspondence to hypometabolism topographies were found at the individual level, independently of the underlying neurodegenerative patterns. Both eTAU and FDG SUVRs significantly distinguished A+/T + from A-/T- individuals (AUC_eTAU_=0.604, AUC_FDG_=0.748) with FDG performing better than eTAU (*p* = 0.04). eAMY and eTAU SUVR showed comparable discriminative power.

**Conclusion:**

Early-phase ^18^F-Flortaucipir-PET can provide perfusion information closely related to brain regional glucose metabolism and perfusion measured by early-phase amyloid-PET, even if less accurate than FDG-PET as a biomarker for neurodegeneration.

**Supplementary Information:**

The online version contains supplementary material available at 10.1007/s00259-024-07063-4.

## Introduction

Alzheimer’s disease (AD) neuropathologic changes are the intracellular oligomers and extracellular accumulation of the β-amyloid (Aβ) protein, the intra-neuron deposition of pathologic tau, and neurodegeneration [1]. These pathological mechanisms begin to accumulate in the brain ten to twenty years before the onset of clinical symptoms. Positron Emission Tomography (PET) imaging can in vivo assess protein deposition and neuronal damage, playing a crucial role in the early diagnosis of AD and other forms of dementia [2]. Brain ^18^F-FDG PET is a well-established technique for studying neurodegeneration by detecting changes in glucose metabolism with distinct patterns that are highly indicative of neurodegeneration across the AD, frontotemporal dementia, and Lewy body spectrum, including individuals in preclinical stages up to clinically full-blown dementia. Moreover, the absence of these disease-specific metabolic patterns strongly predicts preserved cognitive function in longitudinal studies [3].

In addition to providing a measure of protein pathology, there is evidence to suggest that amyloid and tau PET with a dual-phase protocol can capture perfusion information closely related to cerebral glucose metabolism, and early phase images can therefore be used as a surrogate biomarker of neurodegeneration [4]. A dual-phase protocol includes the standard late acquisition of the tracer distribution along with an additional scan performed immediately after the tracer injection. Because of the high lipophilicity of amyloid and tau tracers [4, 5], these early-phase images can provide a proxy for cerebral perfusion that is in turn linked to metabolism based on neurovascular coupling [6]. The early-phase acquisition of amyloid PET has shown a good correlation to ^18^F-FDG PET uptake at the group level [7–19] and the individual level [11], regardless of the amyloid tracer. Indeed, we recently found a comparable distribution of perfusion to that of metabolism at the single-subject level, particularly in the presence of a high neurodegeneration burden in subjects with mild cognitive impairment and dementia [11]. We demonstrated that early phases amyloid-PET imaging can adequately replace ^18^F-FDG-PET imaging in the majority of cases, as they reveal typical neurodegenerative patterns or allow the exclusion of neurodegeneration [11]. Less evidence exists on early phases of tau-PET, despite the similar properties and potential to assess both tau pathology and neurodegeneration. Few studies investigated early phases tau-PET as a potential biomarker of neuronal injury in tauopathies [20] and specifically in AD [14], providing evidence of good correspondence with ^18^F-FDG-PET images and also with early phase of amyloid-PET [21] at the group level. At the individual level, a few case studies assessed and confirmed the comparability of tau early frames and ^18^F-FDG-PET [15, 20]. All previous studies obtained comparable results using different tau tracers, such as ^18^F-PI-2620 and ^18^F-THK5351, but none of them used ^18^F-Flortaucipir which is the most widely applied and the only FDA-approved tracer establishing biomarker evidence of AD tau pathology in living people. Our hypothesis is that ^18^F-Flortaucipir as other tau tracers would be fit similarly to amyloid tracers, given that both have high lipophilicity, for detecting perfusion defects in the early-phase recordings of patients with neurodegenerative diseases.

Our study explored (i) the association of early phase ^18^F-Flortaucipir-PET and ^18^F-FDG-PET and early phase amyloid-PET; (ii) the ability of different modalities to discriminate patients in a memory clinic cohort; (iii) the utility of early phase images of tau-PET for individual patterns’ classification and their comparability with the respective hypometabolic patterns.

## Materials and methods

### Participants

The study included subjects assessed at the Geneva University Hospitals, ranging from cognitively unimpaired (CU) to mild cognitive impairment (MCI) and dementia. The local ethics committee approved the different imaging studies, which were conducted under the principles of the Declaration of Helsinki and the International Conference on Harmonization Good Clinical Practice. All participants signed an informed consent to participate in the study.

We collected all early-phase tau-PET scans performed using ^18^F-Flortaucipir at Geneva University Hospitals (Switzerland) between 2016 and 2023 with the same acquisition protocol. Among them, we selected only subjects for whom at least one 3D T1-weighted MRI scan and an ^18^F-FDG PET scan, acquired within 1 year, were available. This selection resulted in a total of 58 subjects, classified as CU (*n* = 10), MCI (*n* = 34), and dementia (*n* = 14) subjects following standardized clinical criteria [22–24]. A subsample of 36 participants also underwent dual-phase amyloid-PET within 1 year using either [^18^F]florbetapir (eFBP) (*n* = 21) or [^18^F]flutemetamol (eFMM) (*n* = 15). The sample size reached was similar to previous studies’ reporting significant results.

Additionally, we included 43 ^18^F-FDG PET images and 33 early phase tau-PET (eTAU) images of amyloid-negative CU subjects to use as a healthy control (HC) reference for imaging comparisons. Eighteen out of 33 eTAU images were provided by Avid Radiopharmaceuticals/Eli Lilly and Company. For early phase amyloid-PET, we used the previously validated HC group including 28 eFBP and 14 eFMM subjects as published [11].

### MRI acquisition

MRI was performed at Geneva University Hospitals’ division of radiology using a 3-T scanner (Magnetom Skyra, Siemens Healthineers, Erlangen, Germany) equipped with a 20- or 64-channel head coil. The supplemental material, Sect. 1, provides detailed acquisition parameters. The lesion prediction algorithm [25], implemented in the lesion segmentation toolbox, was used to segment fluid-attenuated inversion recovery images, allowing us to extract the total lesion volume. White matter lesions were also rated visually according to the age-related white matter changes scale (ARWMC) [26].

### PET acquisition

PET scans were performed at the Division of Nuclear Medicine and Molecular Imaging at Geneva University Hospitals using a Biograph 128 mCT, Biograph 128 Vision 600 Edge, Biograph 40 mCT, or Biograph 64 TruePoint PET scanner (Siemens Medical Solutions). All scanners’ procedures and image reconstructions were comparable.

Eighteen eTAU images provided by Avid Radiopharmaceuticals/Eli Lilly and Company were acquired within two different studies (Study 1, *N* = 8; Study 2, *N* = 10). In Study 1, the scanning protocol included a dynamic scan 0–60 min and 80–100 min post injection, also including a further dynamic scan at 110–130 min for 3 subjects. Dynamic scans in Study 1 were acquired on either a Siemens Biograph 16 or a GE Discovery VCT, followed by 3D iterative reconstruction (4 iteration / 21 subsets and 3 iteration / 28 subsets, respectively). In Study 2, the scanning protocol included two dynamic scans at 0–60 and 80–130 min post-injection. In Study 2, scans were acquired on either a Philips Gemini or Ingenuity PET/CT, followed by a LOR-RAMLA/BLOB-OS_TF reconstruction. Target injected doses were 370 MBq in Study 1 and 240 MBq in Study 2.

^18^F-FDG PET was performed according to the European Association of Nuclear Medicine guidelines [27]. Tau-PET images were acquired using ^18^F-Flortaucipir. Amyloid-PET images were acquired using ^18^F-florbetapir (FBP) (*n* = 28) or ^18^F-flutemetamol (FMM) (*n* = 14). Regarding the early phase of tau and amyloid-PET acquired at Geneva University Hospitals, image acquisition was started immediately after tracer injection, and a static image was acquired for 5 min (eFBP) or 10 min (eFMM and eTAU) [9, 28]. The supplemental materials, Sect. 2, provide full details on the PET acquisitions.

### MRI and PET normalization processing

Processing was performed as previously described [11] using Statistical Parametric Mapping (SPM 12; Wellcome Trust Centre for Neuroimaging), running in MATLAB R2018b, version 9.5 (MathWorks Inc.). All details are reported in the supplemental materials, Sect. 3.

### SUV ratio (SUVR) extraction in automated anatomic labeling (AAL) ROIs and AD meta–region of interest (Meta-ROI)

Uptake values were extracted within regions from AAL atlas 3 [29] and key regions sensitive to AD according to a predefined meta-ROI approach [30]. An alternative AD-related ROI resembling the AD-typical hypometabolic pattern including larger temporoparietal regions (Figure S1) has been used to extract the SUVR, as a parallel approach to the predefined AD composite meta-ROI created for ^18^F-FDG PET [30]. SUVRs were calculated by normalizing the uptake to the mean value of the pons and cerebellar vermis together as the reference region. Intensity-normalized PET images were saved for further voxel-wise analyses.

### Visual rating and single-subject voxel-wise analyses

Tau status (T+/T-) and amyloid status (A+/A-) were determined for each late image by an expert in nuclear medicine, applying the standard operating procedures approved by the European Medicines Agency. ^18^F-FDG PET, eTAU, and eFBP/eFMM uptake distribution images were visually rated separately and randomly by a nuclear medicine expert blinded to clinical and biomarker information and classified into hypometabolism/hypoperfusion patterns suggestive of neurodegenerative conditions (AD, frontotemporal dementia [FTD], dementia with Lewy bodies [DLB], limbic-predominant age-related TDP-43 encephalopathy [LATE]) or excluding neurodegeneration (negative) [27, 31, 32].

According to a validated SPM single-subject procedure [33], each early-phase PET image and ^18^F-FDG PET image was tested for relative hypometabolism/hypoperfusion by means of a 2-sample t-test in comparison with PET images of controls according to the tracers. This SPM-based single-subject procedure has been demonstrated not to be affected by different scanners used for acquisitions [34]. Control group included 23 ^18^F-FDG PET, 33 eTAU images, 28 eFBP and 14 eFMM of amyloid-negative CU subjects. A published explicit mask was applied to restrict subsequent analyses to within-brain voxels [35]. The statistical threshold for the resulting hypometabolic and hypoperfusion SPM maps was set at a *P* value of 0.05, uncorrected for multiple comparisons, considering significant clusters containing more than 100 voxels. SPM maps were then binarized for further similarity analyses.

### Statistical analysis

Cohen’s kappa (k) was used as a measure of agreement in the assignment of patterns among different modalities (eTAU, eFBP/eFMM, and FDG). Dice coefficient and Simple Matching coefficient (SMC) were calculated, using FSL software [36], to quantify the whole-brain spatial overlap between hypometabolic and hypoperfusion binary maps at the single-subject level (supplemental materials, Sects. 4 and 5) [37]. General linear models were performed to assess the correlation between eTAU, ^18^F-FDG, and eFBP/eFMM SUVR in the AAL ROIs and AD meta-ROI in the whole sample. We assessed the correlations also in T + and T- subjects separately. We tested the correlation of eTAU, eFBP/eFMM, and ^18^F-FDG SUVR in the AD composite meta-ROI with Mini-Mental State Examination (MMSE) scores.

We performed receiver-operating-characteristic (ROC) analyses to compare the discriminative power of eTAU, eFBP, eFMM, and ^18^F-FDG meta-ROIs SUVRs between A-/T- and A+/T + patients. The resulting areas under the curve (AUCs) from different tracers were compared using a De Long test [38] for 2 correlated ROC curves, setting the threshold for significance at a *P* value of 0.05. All statistical analyses were performed with R, version 4.0.2 (R Foundation for statistical computing, https://www.r-project.org/).

## Results

Demographic, clinical, and biomarker data for our cohort are shown in Table 1. The subsamples that underwent an additional early phase amyloid-PET did not significantly differ from the whole sample that underwent eTAU and ^18^F-FDG PET.

**Table 1:** Features of the sample

	Whole sample with eTAU and FDG	sample with eTAU and eFBP	sample with eTAU and eFMM	t statistic subsamples with early amyloid-PET
*N*	58	21	15	
age	70.36 ± 6.5	70.65 ± 6.3	72.25 ± 5.1	0.244
Education, y	13.02 ± 4.3	12 ± 4.7	14 ± 3.7	0.524
gender (F/M)	27/31	9/12	6/9	1
MMSE	24.23 ± 4.8	23.03 ± 5.9	25.79 ± 4.1	0.53
Disease Stage (CU/MCI/DEM)	10/34/14	5/10/6	1/12/2	0.138
Tau status -/+	26/31	11/9	6/9	0.591
Global Tau SUVR	1.41 ± 0.35	1.4 ± 0.34	1.4 ± 0.33	0.771
Amyloid status +/-	37/18	7/13	4/11	0.875
Centiloid	53 ± 48	55 ± 55	61 ± 48	0.764

Amyloid status and Tau status are based on PET visual reading

Abbreviations: Amy = amyloid; CU = cognitively unimpaired; DEM = dementia; eTAU = early-phase of tau PET; eFBP = early-phase of ^18^ F-Florbetarpi PET; eFMM = early-phase of ^18^ F-Flutametamol; F = females; M = males; MCI = Mild Cognitive Impairment; MMSE = Mini-Mental State Examination; N = number; SUVR = Standardized uptake value; y = year

### Correlations between eTAU, 18F-FDG, and eFBP/eFMM SUVR

**Fig. 1:**
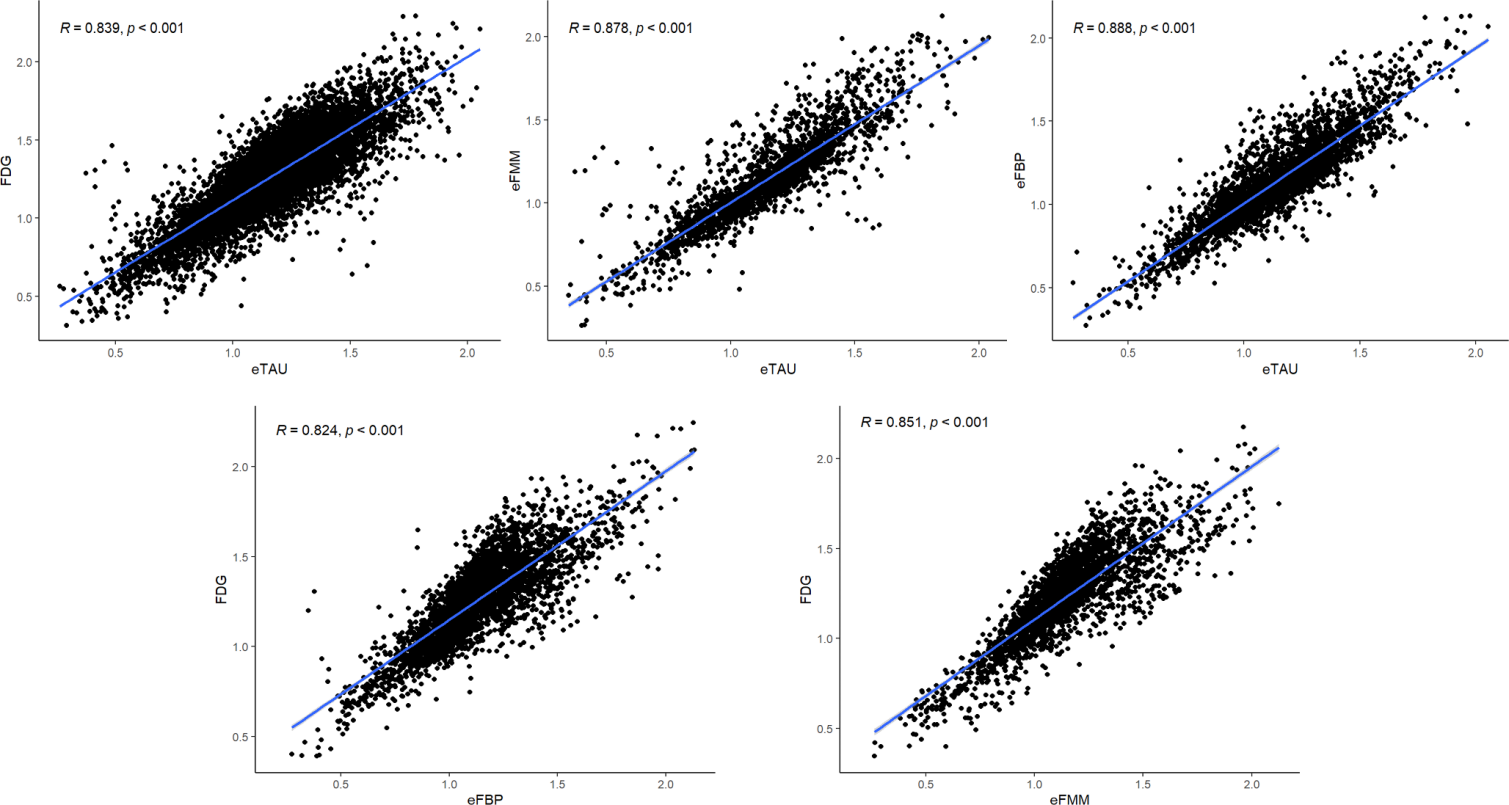
Correlation of eTAU SUVR, ^18^F-FDG-PET SUVR and eFPB/eFMM SUVR. Scatterplots in the upper row show associations between eTAU (x-axis) in AAL regions and their respective ^18^F-FDG SUVR (y-axis) or eFBP/eFMM SUVR (y-axis). Scatterplots in the row below show associations between eFBP/eFMM SUVR (x-axis) in AAL regions and their respective ^18^F-FDG SUVR (y-axis). Results are presented for the whole sample. Lines resulting from linear regression are shown in blue. R and *P* values are given in the upper left corner. FBP = florbetapir; FMM = flutemetamol; eFBP = early FBP; eFMM = early FMM; eTAU = early tau PET

eTAU SUVRs in the AAL ROIs showed a strong correlation with ^18^F-FDG SUVRs in the whole group (*r* = 0.839, *p* < 0.001), and in T+ (*r* = 0.855, *p* < 0.001) and T- (*r* = 0.834, *p* < 0.001) subgroups when considered separately. Strong correlations were found between eTAU SUVRs and early-phase amyloid-PET SUVRs, regardless of the amyloid tracer (*r* > 0.87, *p* < 0.001). Early-phase amyloid-PET SUVRs also showed a strong association with their respective ^18^F-FDG SUVRs, independently from the amyloid tracers (*r* > 0.82, *p* < 0.001). Figure 1 shows scatterplots for the whole sample. When we consider the SUVRs in the AD meta-ROI, the correlations of eTAU with the other modalities were still significant and high (*r* > 0.71, *p* < 0.001), as well as the correlations between early-phase amyloid-PET and ^18^F-FDG SUVRs (eFMM *r* = 0.88, *p* < 0.001; eFBP *r* = 0.62, *p* < 0.001).

The composite meta-ROI SUVRs for eTAU, eFBP/eFMM, and ^18^F-FDG uptake correlated significantly with MMSE scores (eTAU *r* = 0.441, *p* < 0.001; ^18^F-FDG *r* = 0.559, *p* < 0.001; eAMY *r* = 0.486, *p* = 0.003).

### Discriminative performance of AD Meta-ROI approaches

**Fig. 2:**
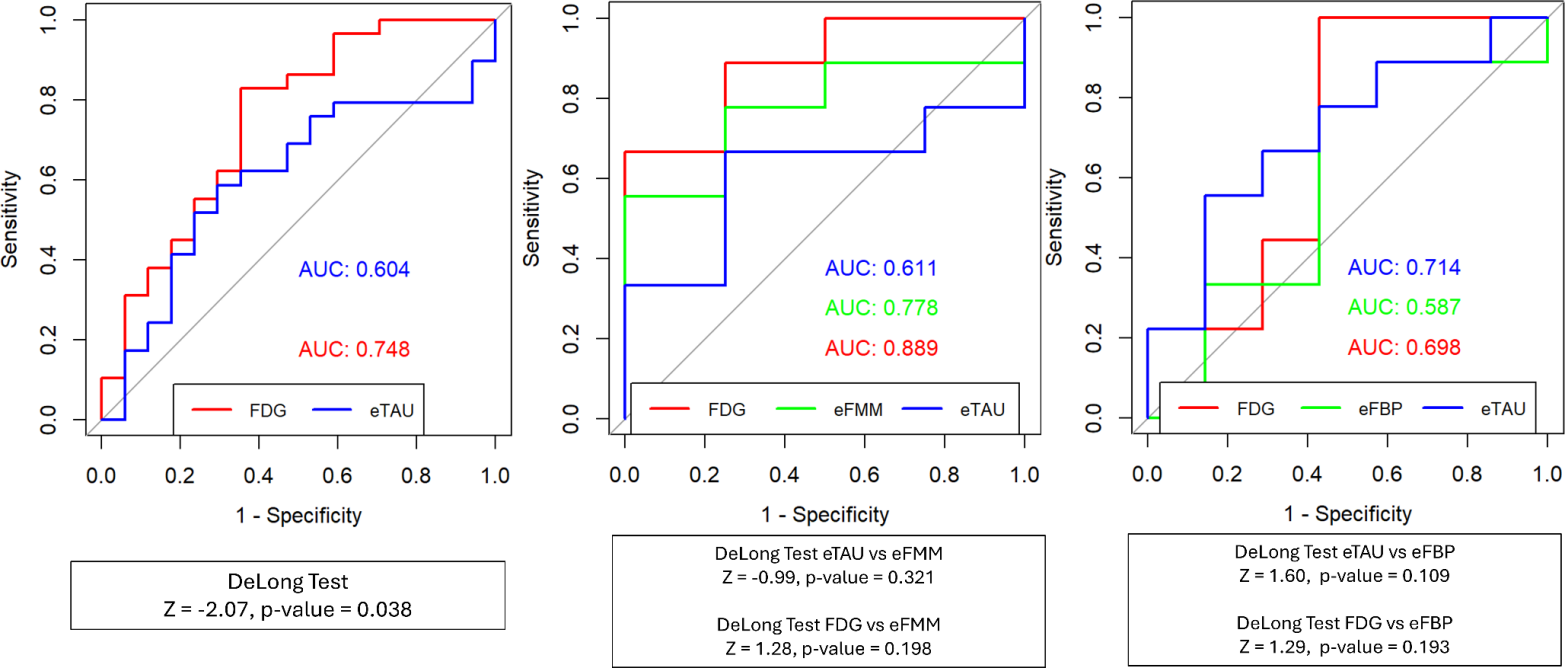
Discriminative performance of eTAU and ^18^F-FDG, and eFBP/eFMM SUVR. ROC shows the diagnostic performance of SUVR in AD composite meta-ROI for distinguishing A+/T + patients from A-/T-

When testing the performance of the eTAU SUVRs in the AD composite meta-ROI [30] in distinguishing A+/T+ (*n* = 30 in the whole sample) from A-/T- (*n* = 18 in the whole sample) subjects, we found good AUC discriminative value (AUC = 0.604), as for the eFMM/eFBP SUVRs (AUC_eFMM_=0.611; AUC_**e**FBP_=0.587), but slightly lower than ^18^F-FDG SUVR AUC (AUC = 0.748). The DeLong test confirmed a better discriminative performance of ^18^F-FDG (*p* = 0.04) as compared with eTAU and no significant differences in the discriminatory performance of eTAU and eFBP/eFMM (*p* > 0.05). ^18^F-FDG PET SUVR did not show a significantly better discriminative performance than eFBP/eFMM SUVR (*p* > 0.05). Figure 2 compares the diagnostic performance of eTAU SUVRs, ^18^F-FDG PET SUVRs, and eFBP/eFMM SUVRs in the AD composite meta-ROI in terms of ROC curves. Comparable results were obtained using the alternative AD meta-ROI.

### Individual patterns of hypometabolism and hypoperfusion

**Fig. 3:**
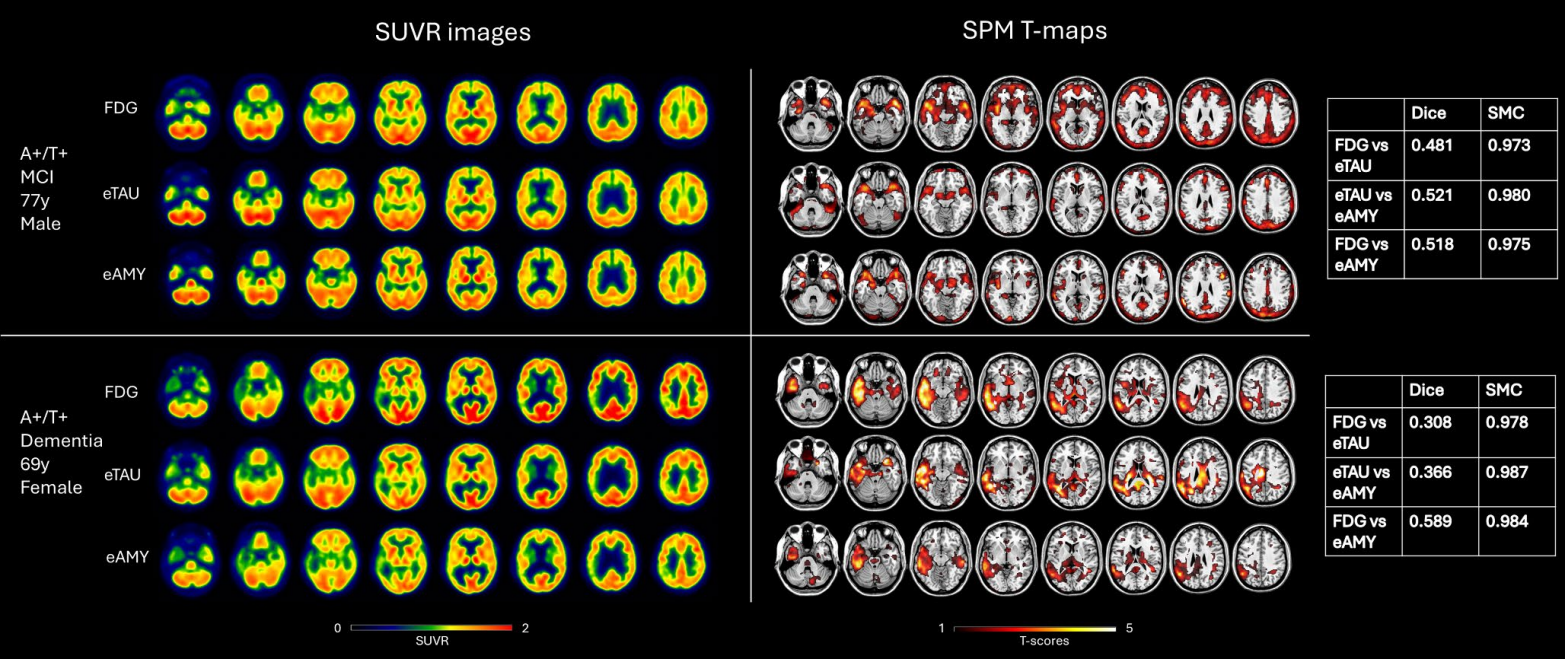
Individual examples of hypometabolic and hypoperfusion standardized uptake distribution images and SPM t-maps. On the left, axial views of representative [^18^F]FDG-PET SUVR, early-phase tau-PET SUVR, early-phase amyloid-PET SUVR images in a patient with prodromal AD (MCI, top row) and in a patient with AD dementia (bottom row). On the right, in the same order, the SPM single-subject T-maps of the same subjects are displayed on the standard Montreal Neurological Institute template. The tables showed the similarity coefficients (Dice and simple matching coefficient (SMC)). A = amyloid; eAMY = early phase amyloid-PET; eTAU = early phase tau-PET; MCI = mild cognitive impairment; T = tau

**Fig. 4:**
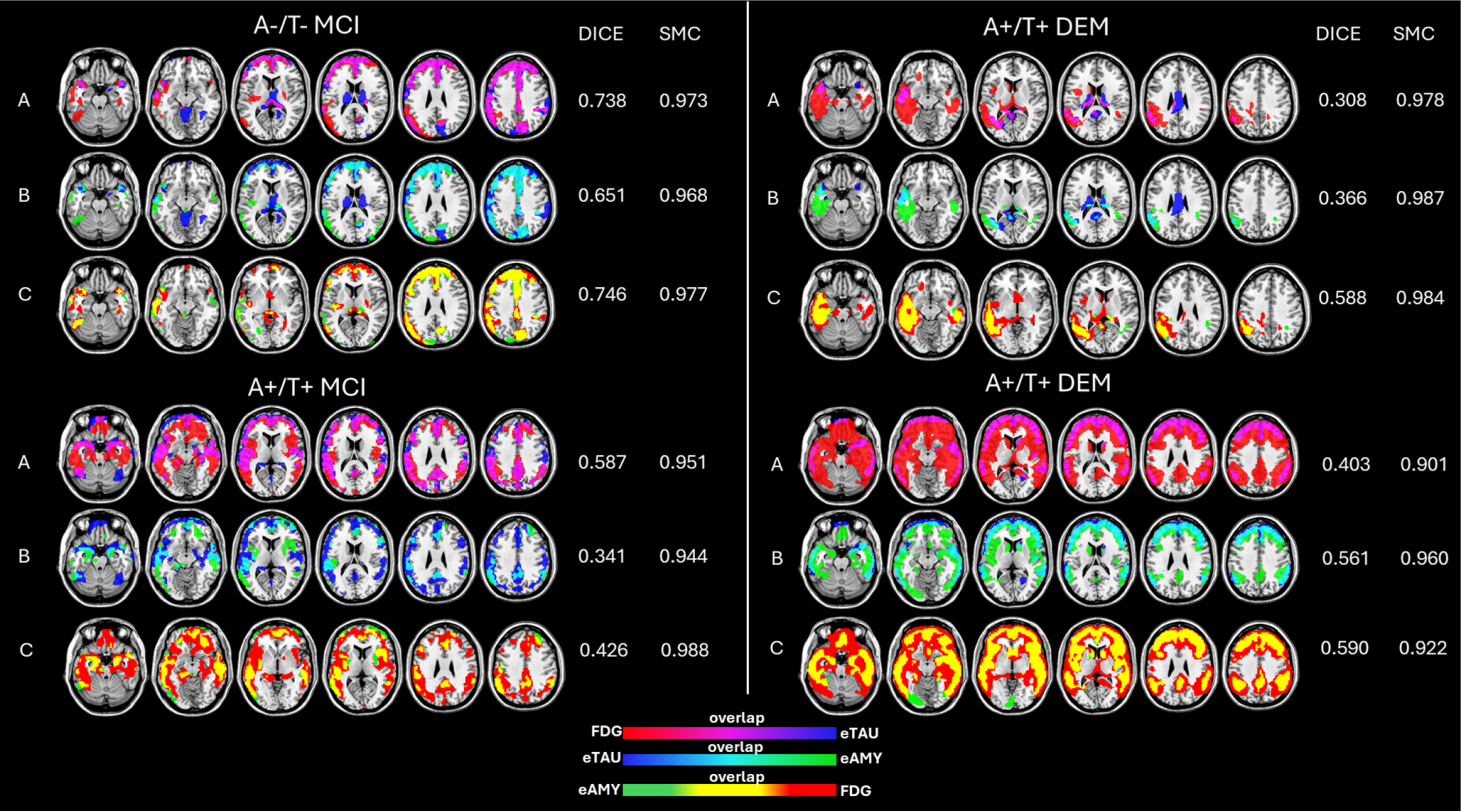
Individual examples of hypometabolic and hypoperfusion patterns at the single-subject level. Hypometabolism maps, two hypoperfusion maps (eTAU and eAMY), and their overlap were imposed on standard Montreal Neurological Institute template. These maps were obtained from the binarization of single-subject eTAU SPM T-maps, ^18^F-FDG PET SPM T-maps, and eAMY SPM T-maps (*p* < 0.05 uncorrected, k > 100). The Dice similarity index and the Simpe Matching coefficient (SMC) are reported to the right of the brain template of each subject for each modalities’ comparison. For each individual case, three rows (**A**, **B**, **C**) show the topographical comparison between eTAU and ^18^F-FDG PET (**A**), between eTAU and eAMY (**B**), and between ^18^F-FDG PET and eAMY (**C**). The reported individual cases are at different clinical disease stages and have different biomarker status. The first case (left) is A-/T- and shows FTD-like patterns. The other cases are all A+/T + with AD-like patterns. A = amyloid; AD = Alzheimer’s disease; DEM = dementia; eAMY = early phase amyloid-PET; eTAU = early phase tau-PET; FTD = frontotemporal dementia; MCI = mild cognitive impairment; SMC = simple matching coefficients; T = tau

The visual rating confirmed disease-specific hypometabolism and hypoperfusion patterns (Figs. 3 and 4, Table S1). The rating of ^18^F-FDG-PET images allowed the identification of different neurodegenerative patterns: temporoparietal hypometabolism (AD-like pattern, *n* = 22), frontotemporal hypometabolism (FTD-like pattern, *n* = 4), and temporoparietal and occipital hypometabolism (DLB-like pattern, *n* = 2). Of note, 24 of 58 subjects showed negative ^18^F-FDG images for neurodegenerative patterns and 6 out of 58 images resulted unclassifiable for any specific patterns. Our analysis revealed good agreement between the classifications of hypometabolic and eTAU hypoperfusion images (k = 0.58, *p* < 0.001). For 70.6% of subjects, the rating by ^18^F-FDG PET was consistent with the one using early-phase tau-PET images. 79% of negative ^18^F-FDG scans were negative also at eTAU rating, whereas the remaining were unclassifiable. The frequency of the different hypometabolism and eTAU hypoperfusion patterns is reported in Table S1. A good agreement was also found between the classifications of hypoperfusion images obtained with eTAU and eFBP/eFMM (k = 0.60, *p* < 0.001). For 72% of subjects, the rating by eTAU was consistent with the one with eFBP/eFMM. The frequency of the different eTAU and eFBP/eFMM hypoperfusion patterns is reported in **Table S2**. Our analysis on the subsample with ^18^F-FDG-PET and early-phase of amyloid-PET confirmed a good agreement between the classifications of hypometabolic and eFBP/eFMM hypoperfusion images (k = 0.71, *p* < 0.001) (**Table S3**). Notably, 93% of negative ^18^F-FDG scans were negative also at eAMY rating.

Table 2 shows the frequency of hypometabolism patterns and their spatial overlaps with eTAU hypoperfusion maps as measured by the rating, Dice, and SMC in the whole sample and separately in the 3 clinical subgroups (CU, MCI, and dementia). The SMCs were very high (all > 0.9) indicating strong similarities between hypoperfusion and hypometabolism images, when considering the overall proportion of concordance (both pathological and normal voxels between different modalities). The SMC indicated higher overlap than the Dice that, as a metric, gives no credit to concordant normal voxels. Also, the visual match was higher overall than the Dice scores, likely because Dice considers small hypometabolic/hypoperfusion spots as irrelevant for the clinical rating. Regarding the clinical staging, the match tends to be higher for demented cases, followed by MCI and CU. The results obtained by the same analyses comparing hypometabolism and eAMY hypoperfusion patterns are reported in the supplementary materials, Table S4. Whereas the SMC are similarly high regardless of the considered modalities, the Dice scores between these two latter modalities (eAMY and ^18^F-FDG) seem higher than the ones of hypometabolic vs. eTAU patterns and higher for demented and MCI cases.

**Table 2:** Distribution of hypometabolism patterns and Voxel-by-Voxel concordance with Hypoperfusion Patterns obtained with early phase of tau PET in clinical groups

Hypometabolic patterns	Whole sample				Dementia				MCI				CU			
	Sample (*N* = 58)	SMC	Dice	% match	Sample (*N* = 14)	SMC	Dice	% match	Sample (*N* = 34)	SMC	Dice	% match	Sample (*N* = 10)	SMC	Dice	% match
AD-like	22	0.97 ± 0.02	0.3 ± 0.2	68%	9	0.96 ± 0.03	0.3 ± 0.1	88%	12	0.97 ± 0.01	0.4 ± 0.2	58%	1	0.99	0	0%
FTD-like	4	0.96 ± 0.02	0.3 ± 0.2	75%	1	0.95	0.6	100%	3	0.97 ± 0.03	0.2 ± 0.2	66%	0	/	/	/
DLB-like	2	0.99 ± 0.01	0.3 ± 0.4	0%	0	/	/	/	2	0.99 ± 0.05	0.3 ± 0.4	0%	0	/	/	/
Unclassified	6	0.98 ± 0.02	0.2 ± 0.2	66%	2	0.97 ± 0.03	0.5 ± 0.1	50%	3	0.98 ± 0.01	0.4 ± 0.1	66%	1	0.99	0.1	100%
Normal	24	0.98 ± 0.02	0.3 ± 0.3	79%	2	0.98 ± 0.02	0.5 ± 0.1	0%	14	0.98 ± 0.02	0.4 ± 0.3	86%	8	0.99 ± 0.08	0.1 ± 0.1	87%

Abbreviations: AD = Alzheimer disease; FTD = frontotemporal dementia; DLB = dementia with Lewy bodies; MCI = mild cognitive impairment; CU = cognitively unimpaired; SMC = simple matching coefficient

Seventeen of 58 subjects (29.3%) showed a mismatch between eTAU and ^18^F-FDG scans, whereas 10 of 36 (27%) between eTAU and eFBP/eFMM scans. Match and mismatch subjects did not differ in any demographic variables, namely age, gender, MMSE, diagnostic stages, amyloid, and tau loads (*p* > 0.05). When we compared MRI total lesion volume and ARWMC scores between the matched and mismatched subgroups based on eTAU and ^18^F-FDG scans, we did not find differences in ARWMC (*p* = 0.12) and total lesion volume (*p* = 0.14). When we compared MRI total lesion volume and ARWMC scores between the matched and mismatched subgroups based on eTAU and eFBP/eFMM scans, we found more severe cerebrovascular pathology based on ARWMC in matched cases that mismatched (*p* = 0.02), but not using the total lesion volume (*p* = 0.75).

## Discussion

This study assessed the association of early frames of ^18^F-Flortaucipir PET and amyloid-PET, and ^18^F-FDG-PET and their comparability in discriminating patients in a memory clinic setting. Our findings indicate that early phases ^18^F-Flortaucipir PET can provide perfusion information closely related to brain regional glucose metabolism and even more to perfusion measured by early phases amyloid-PET. Early phases amyloid-PET have been well established as a good neurodegeneration biomarker with the capability to substitute for ^18^F-FDG-PET [11]. Given the general relationship between perfusion and energy metabolism in AD [39], dual-phase tau PET may also provide a surrogate for ^18^F-FDG-PET information indicative of neurodegeneration. In this way, it would permit assessing the topographical distribution of neurodegeneration and tau pathology with a single tracer injection, with less cost and radiation exposure. Information on tau and neurodegeneration status are also highly indicative of amyloid status since the majority of T+/N + subjects are also A+ [1]. Here, all T+/N + subjects identified by dual-phase ^18^F-Flortaucipir PET were A+. A recent study also supported the beneficial role of incorporating cortical hypoperfusion as a biomarker of neuro injury in cases with ambiguous AD profile and promising trends in 4R-tauophaties suggest further investigation in other neurodegenerative diseases [40].

In line with previous studies [14, 15, 20], we confirmed a strong correlation between eTAU and ^18^F-FDG SUVR (*r* = 0.839, *p* < 0.001) in a memory clinic sample, regardless of the tau status. Our group-level results confirmed the correlation between metabolism and perfusion measured with the only FDA-approved tau tracer (^18^F-Flortaucipir) by replicating the results previously obtained with second-generation tau tracers and dynamic acquisition protocols [14, 15, 20]. The correlation coefficients were comparable to those obtained from the correlations between eFMM/eFBP and ^18^F-FDG SUVR (*r* > 0.824, *p* < 0.001) suggesting the comparability between the two perfusion measures as surrogates for ^18^F-FDG-PET. A strong equivalence of eTAU and eAMY was further confirmed by direct correlations between their SUVRs (*r* > 0.878, *p* < 0.001). Interestingly, a recent study similarly demonstrated the comparability of ^18^F-PI-2620-tau-PET and eFMM as surrogates of cerebral perfusion deficit with robust agreement, especially in disease-specific signature regions [21]. Similarly, the relative cerebral blood flow derived from dynamic PET can be used interchangeably regardless of the choice of FBP or ^18^F-flortaucipir tracer [41]. We additionally found both perfusion and metabolism measures in AD-related regions significantly correlated with MMSE, in line with the close relationship between neurodegeneration and cognition [42].

Accordingly, eTAU perfusion measured in AD-specific regions showed a good diagnostic performance in discriminating A+/T + individuals from A-/T-, however, without reaching the same performance of ^18^F-FDG-PET (*p* = 0.038). The DeLong test did not show differences in the discriminative performance of eAMY and eTAU confirming the comparability of the two perfusion measures. Notably, the discriminative performance of ^18^F-FDG-PET did not differ significantly from eAMY, in line with our previous study [11]. Some explanations of the slightly better performance of eAMY than eTAU when compared to ^18^F-FDG-PET may be driven by tracers’ specific properties, such as differences in the lipophilicity, nonuniform delivery and unidirectional influx of the tracers in the brain. Moreover, AD-relevant regions are well-validated for ^18^F-FDG PET [30], instead, early-phase images are, in this respect, less standardized. For this reason, the same discriminative analyses were also run using an alternative large AD meta-ROI, obtaining equivalent results. Also, the reference regions choice was aligned with standard one used for ^18^F-FDG-PET to ensure a robust comparison, and other reference regions can be employed; thus, further studies may assess the best reference regions specifically for early-phase images [15].

To define whether early phases of tau-PET can replace ^18^F-FDG-PET for individual classification in clinical practice, we classified perfusion and metabolic images as neurodegenerative patterns or negative scans. Our results demonstrated a fair agreement between reduced perfusion in eTAU and reduced glucose metabolism in ^18^F-FDG-PET leading to the same classification in most cases (70.6%). Out of 24 subjects characterized as negative scans by ^18^F-FDG-PET (mostly CU and MCI), the majority (*n* = 19) were correctly classified as negative also with eTAU suggesting a similar ability in excluding the presence of neurodegeneration. In the case of neurodegenerative ^18^F-FDG-PET patterns, characterizing mainly individuals with MCI and dementia, the rating of eTAU led to the same rating for 64% of subjects with a higher frequency of match in dementia than MCI. The strong similarities between the images were also supported by the high SMC suggesting good concordance in both pathological and normal voxels at the single-subject level. Despite the good visual match and the high SMC, the Dice scores that consider mainly the pathological voxels indicated a low degree of overlap of eTAU with hypometabolic maps. A possible explanation is the noisy feature of the initial frames and the nonuniform delivery of the tracer [13], which may result in significant spots in the hypoperfusion maps. The imperfect semiquantitative overlap at Dice also characterized the comparison between eAMY and ^18^F-FDG-PET as the gold standard, even if the Dice scores were higher than those of eTAU vs. ^18^F-FDG-PET and ratings of eAMY led to the same neurodegenerative patterns’ classification for 82% of pathological cases at ^18^F-FDG-PET. In our previous study comparing eAMY and ^18^F-FDG-PET in a large sample of 166 individuals [11], we found, in general, a good degree of overlap as measured by Dice that increased with the clinical severity. For this reason, we suggested an additional ^18^F-FDG-PET in preclinical and prodromal disease stages when the neurodegeneration may be not severe enough to be detected by perfusion measures. The lack of a full overlap can be partially explained by the different brain biological processes measured by the two modalities [17, 43]. Moreover, this discrepancy paves the way for further studies exploring different early time frames for ^18^F-Flortaucipir. To our knowledge, few previous studies have already used dynamic ^18^F-Flortaucipir PET to assess cerebral blood flow in AD [41, 44], using an initial scanning protocol of 130 min, consisting of a 60-min dynamic emission scan or a shortened scanning protocol of 100 min [45]. However, these studies did not address the need to assess the optimal early time frame of ^18^F-Flortaucipir in association with ^18^F-FDG-PET or ^15^O-H_2_O PET, as it has been done for some amyloid tracers [16, 18].

When we focused on individuals with a visual mismatch between hypometabolism and eTAU hypoperfusion (29.3%), we couldn’t find any differences in the demographic variables compared to individuals with a match between the modalities’ classification. Contrary to our hypothesis of eTAU as more sensitive to atrophy and cerebrovascular factors than ^18^F-FDG-PET [6], the mismatched group did not show greater cerebrovascular lesion volumes at MRI than the match group. Instead, greater white matter lesions characterized individuals with a match between the two perfusion measures than a mismatch, suggesting that both eTAU and eAMY suffer from biases due to the presence of cerebrovascular diseases similarly [6].

The potential limitations of this study arise from the time frames used for eTAU, here acquired in a clinical setting using published protocols based on different tracers [9]. Further studies using dynamic acquisition to explore different time frames for ^18^F-Flortaucipir to achieve the best association with ^18^F-FDG-PET are largely encouraged. Another limiting factor in this study is the small sample size of HC used for comparison to obtain the maps for the similarity analyses. Public dataset of normal controls for early phase imaging represents an important need to obtain a good performance in voxel- wise analyses and, in this respect, public sources for early-phase images are less mature than for ^18^F-FDG PET. Moreover, we acknowledge that our sample is mainly composed of subjects in AD spectrum; indeed, generalizability of the findings for patterns of other neurodegenerative diseases needs to be further explored in large samples.

## Conclusion

This study assessed for the first time the comparability of early phase ^18^F-Flortaucipir PET with ^18^F-FDG PET as the gold standard, and the early phase of amyloid-PET as another perfusion measure. The strong group-level correlations and the good concordance in classifying different neurodegenerative patterns (or excluding them) support the use of early phase ^18^F-Flortaucipir PET as a measure of neurodegeneration closely associated with brain regional glucose metabolism. Therefore, dual-phase tau-PET represents a promising protocol to assess both tau pathology and neurodegeneration as surrogate biomarker with a single tracer injection, with advantages in terms of costs, patient comfort, and radiation exposure in clinical practice.

## Electronic supplementary material

Below is the link to the electronic supplementary material.

Supplementary Material 1

## Data Availability

Anonymized data used in this study are available upon reasonable request from the corresponding author.
