# Supplementary material for: Early-phase 18F-Flortaucipir tau-PET as a proxy of brain metabolism in Alzheimer’s disease: a comparison with 18F-FDG-PET and early-phase amyloid-PET
Source: Eur J Nucl Med Mol Imaging. 2025 Jan 24;52(6):1958–69. doi: 10.1007/s00259-024-07063-4 (PMC12014785; doi:10.1007/s00259-024-07063-4)
Supplement: Supplementary file 1 — Supplementary Material 1 [file 259_2024_7063_MOESM1_ESM.docx]

**Supplementary Materials**

**1. MRI acquisition**

The following acquisition parameters were used: repetition time [TR] = 1930 ms, echo time [TE] = 2.36 msec, field of view = 256 x 256 mm, flip angle = 8°, slice thickness = 0.9 mm, matrix size = 288 x 288 pixels. Or a high-resolution anatomical 3D T1 was acquired on a 3 Tesla MR750w scanner (GE Healthcare, Milwaukee, Wisconsin) with the following parameters: matrix size = 254 x 254, slices = 178, 1mm isotropic, TR = 7.2 ms.

**2. PET acquisition**

All scanners were from the same vendor and of the same generation, harmonized regarding their performance and reconstructions, and cross-calibrated.

*^18^F-FDG-PET* - ^18^F-FDG-PET was performed according to the European Association of Nuclear Medicine (EANM) guidelines (*1*,*2*). Subjects fasted for at least 4 hours. Before radiopharmaceutical injection, blood glucose was checked and was 7mmol/L or less for all subjects. Subjects were injected with 203.89±15.62 MBq of ^18^F-FDG via a venous cannula, eyes open in a dimly lit room. The required minimum time interval between injection and scan start was 30 minutes. The scanning time lasted 20 minutes. Data were acquired in list mode and were reconstructed using 3D OSEM 6 interactions 8 subsets, and a 2mm Gaussian filter at Full Width and Half Maximum, resulting in images with 400x400 matrix with 1.01mm isotropic voxels.

*Amyloid-PET* - ^18^F-FBP late images were acquired 50 min after the intravenous administration of 210±18.77 MBq (3 × 5-min image frames). ^18^F-FMM late images were acquired 90 min after the intravenous administration of 166±16.73 MBq (4 × 5-min image frames). Images were then averaged into a single 15- or 20-minutes frame. All amyloid-PET late images were visually assessed by an independent, board-certified specialist in nuclear medicine (VG) applying the standard operating procedures approved by the European Medicines Agency ([https://www.ema.europa.eu/documents/product-information/vizamyl-epar productinformation_en.pdf](https://www.ema.europa.eu/documents/product-information/vizamyl-epar%20productinformation_en.pdf);<https://www.ema.europa.eu/documents/product-information/amyvid-epar-product-information_en.pdf> ). Subjects were classified based on the visual inspection of the late images into "A+” or "A-” (i.e., subjects that presented high levels and low levels of cortical amyloid binding, respectively).

*Tau-PET* - ^18^F-flortaucipir (18F-AV1451), synthesized at the Center for Radiopharmaceutical Sciences in ETH Zurich, Switzerland, under license from the intellectual property (IP) owner (Avid subsidiary of Lilly, Philadelphia, PA, USA), was used for the tau-PET scans. Subjects received 180 MBq of ^18^F-AV1451, with late image acquisition performed 75 min after injection (acquisition time 30 min) (*3*). Each emission frame was reconstructed in 6 × 5 min frames. Subjects were classified based on the visual inspection of the late images into "T+” or "T-” according to published recommendations (*4*,*5*) (i.e., subjects that presented high levels and low levels of tau binding, respectively).

Data were acquired in list mode and were reconstructed using 3D OSEM 4 interactions 8 subsets, and a 2mm Gaussian filter at Full Width and Half Maximum, resulting in images with 400x400 matrix with 1.01mm isotropic voxels.

**3. MRI and PET normalization processing**

MRI 3D T1 sequences were aligned to a reference plane passing through the anterior commissure, segmented into gray matter, white matter, and cerebrospinal fluid tissue compartments, and normalized to the Montreal Neurologic Institute (MNI) space using tissue probability maps. ^18^F-FDG, eTAU, and eFBP/eFMM images were aligned to the subject’s respective T1 MRI image and normalized to the MNI space using the transformation matrix that was generated during the registration of the MRI images to the standard space. PET images were spatially smoothed with an isotropic 3D 8mm Gaussian kernel (Full Width and Half Maximum).

**4. Dice similarity coefficient**

We compared the resulting hypometabolism and hypoperfusion maps using the Dice as a measure of concordance. Dice coefficient for binary maps A and B is defined as:

$$Dice=\frac{2*\left( A\cap B \right)}{A+B}$$

It takes the value of 1 if A and B assume the same logical value in every pixel (high concordance), and a value of 0 if they always disagree (null concordance). It is interpreted as follows: <0.2, poor; 0.2–0.4, fair; 0.4–0.6, moderate; 0.6–0.8, good; and >0.8, excellent agreement (*6*).

**5. Simple Matching coefficient**

The Simple Matching Coefficient (SMC) is a similarity measure between two binary images. The SMC is calculated as:

$$SMC=\frac{a+d}{a+b+c+d}$$

where: 𝑎 is the number of voxels where both images have a value of 1; 𝑏 is the number of voxels where the first image has a value of 1 and the second image has a value of 0; 𝑐 is the number of voxels where the first image has a value of 0 and the second image has a value of 1; 𝑑 is the number of voxels where both images have a value of 0. The SMC ranges from 0 to 1. If the SMC is close to 1, it suggests that the two images are very similar, sharing a high proportion of matching voxels (both 1s and 0s); if the SMC is close to 0, it suggests that the two images are quite different, with very few matching voxels.

**Table S1.** Contingency Table Reporting Frequency of Different Hypometabolism and Hypoperfusion Patterns Obtained with Early Phase of Tau PET in Whole Sample

| **eTAU hypoperfusion patterns** | | | | | | | |
| --- | --- | --- | --- | --- | --- | --- | --- |
| **Hypometabolic patterns** | **AD-like** | **FTD-like** | **DLB-like** | **Limbic-like** | **Unclassified** | **Normal** | **Total** |
| AD-like | 15 | 0 | 0 | 0 | 3 | 4 | 22 |
| FTD-like | 0 | 3 | 0 | 1 | 0 | 0 | 4 |
| DLB-like | 1 | 0 | 0 | 0 | 1 | 0 | 2 |
| Limbic-like | 0 | 0 | 0 | 0 | 0 | 0 | 0 |
| Unclassified | 2 | 0 | 0 | 0 | 4 | 0 | 6 |
| Normal | 0 | 1 | 0 | 0 | 4 | 19 | 24 |
| Total | 18 | 4 | 0 | 1 | 12 | 23 | 58 |
| AD=Alzheimer disease; FTD=frontotemporal dementia; DLB =dementia with Lewy bodies; eTAU= early-phase of tau-PET | | | | | | | |

**Table S2.** Contingency Table Reporting Frequency of Different Hypoperfusion Patterns Obtained with Early Phase of Tau and Amyloid PET in Whole Sample

|  | **eTAU hypoperfusion patterns** | | | | | |  |
| --- | --- | --- | --- | --- | --- | --- | --- |
| **eAMY hypoperfusion patterns** | **AD-like** | **FTD-like** | **DLB-like** | **Limbic-like** | **Unclassified** | **Normal** | **Total** |
| AD-like | 10 | 0 | 0 | 0 | 3 | 0 | 13 |
| FTD-like | 1 | 2 | 0 | 0 | 0 | 0 | 3 |
| DLB-like | 0 | 0 | 0 | 0 | 1 | 0 | 1 |
| Limbic-like | 0 | 0 | 0 | 0 | 0 | 0 | 0 |
| Unclassified | 0 | 0 | 0 | 0 | 1 | 1 | 2 |
| Normal | 0 | 0 | 0 | 0 | 4 | 13 | 17 |
| Total | 11 | 2 | 0 | 0 | 9 | 14 | 36 |
| AD=Alzheimer disease; FTD=frontotemporal dementia; DLB =dementia with Lewy bodies; eTAU= early-phase of tau-PET; eAMY= early-phase of amy-PET | | | | | | | |

**Table S3.** Contingency Table Reporting Frequency of Different Hypometabolism and Hypoperfusion Patterns Obtained with Early Phase of Amyloid PET in Whole Sample

|  | **eAMY hypoperfusion patterns** | | | | | |  |
| --- | --- | --- | --- | --- | --- | --- | --- |
| **Hypometabolic patterns** | **AD-like** | **FTD-like** | **DLB-like** | **Limbic-like** | **Unclassified** | **Normal** | **Total** |
| AD-like | 11 | 1 | 0 | 0 | 0 | 1 | 13 |
| FTD-like | 0 | 2 | 0 | 0 | 0 | 0 | 2 |
| DLB-like | 1 | 0 | 1 | 0 | 0 | 0 | 2 |
| Limbic-like | 0 | 0 | 0 | 0 | 0 | 0 | 0 |
| Unclassified | 1 | 0 | 0 | 0 | 1 | 2 | 4 |
| Normal | 0 | 0 | 0 | 0 | 1 | 14 | 15 |
| Total | 13 | 3 | 1 | 0 | 2 | 17 | 36 |
| AD=Alzheimer disease; FTD=frontotemporal dementia; DLB =dementia with Lewy bodies; eTAU= early-phase of tau-PET; eAMY= early-phase of amy-PET | | | | | | | |

**Table S4.** Distribution of Hypometabolism Patterns and Their Voxel-by-Voxel Concordance with Hypoperfusion Patterns obtained with Early Phase of Amyloid PET in Clinical Groups

|  | **Whole sample** | | | | **Dementia** | | | | **MCI** | | | | **CU** | | | |
| --- | --- | --- | --- | --- | --- | --- | --- | --- | --- | --- | --- | --- | --- | --- | --- | --- |
| **Hypometabolic patterns** | **Sample (N=36)** | **SMC** | **Dice** | **% match** | **Sample (N=8)** | **MSC** | **Dice** | **% match** | **Sample (N=22)** | **SMC** | **Dice** | **% match** | **Sample (N=6)** | **SMC** | **Dice** | **% match** |
| **AD-like** | 13 | 0.97 ± 0.02 | 0.5 ± 0.1 | 76% | 5 | 0.96 ± 0.03 | 0.5 ± 0.1 | 80% | 8 | 0.97 ± 0.01 | 0.5 ± 0.1 | 75% | 0 | / | / | / |
| **FTD-like** | 3 | 0.97 ± 0.02 | 0.6 ± 0.1 | 66% | 1 | 0.95 | 0.5 | 100% | 2 | 0.98 ± 0.01 | 0.6 ± 0.2 | 50% | 0 | / | / | / |
| **DLB-like** | 1 | 0.99 | 0.5 | 0% | 0 | / | / | / | 1 | 0.99 | 0.5 | 0% | 0 | / | / | / |
| **Unclassified** | 2 | 0.98 ± 0.01 | 0.6 ± 0.1 | 50% | 0 | / | / | / | 2 | 0.97 ± 0.01 | 0.6 ± 0.1 | 50% | 0 | / | / | / |
| **Normal** | 17 | 0.98 ± 0.02 | 0.3 ± 0.1 | 76% | 2 | 0.99 ± 0.01 | 0.5 ± 0.1 | 0% | 9 | 0.98 ± 0.02 | 0.3 ± 0.2 | 88% | 6 | 0.99 ± 0.01 | 0.2 ± 0.1 | 83% |
| AD=Alzheimer disease; FTD=frontotemporal dementia; DLB =dementia with Lewy bodies; MCI=mild cognitive impairment; CU=cognitively unimpaired, SMC=simple matching coefficient | | | | | | | | | | | | | | | | |

**Figure S1. Alternative meta-ROI for AD-related pattern.** The meta-ROI is based on previous literature resembling the Alzheimer’s disease-typical hypometabolic pattern including temporoparietal regions and has been used to extract the SUVR for early-phase perfusion and hypometabolic images as an alternative and parallel approach to Landau’s AD composite meta-ROI.


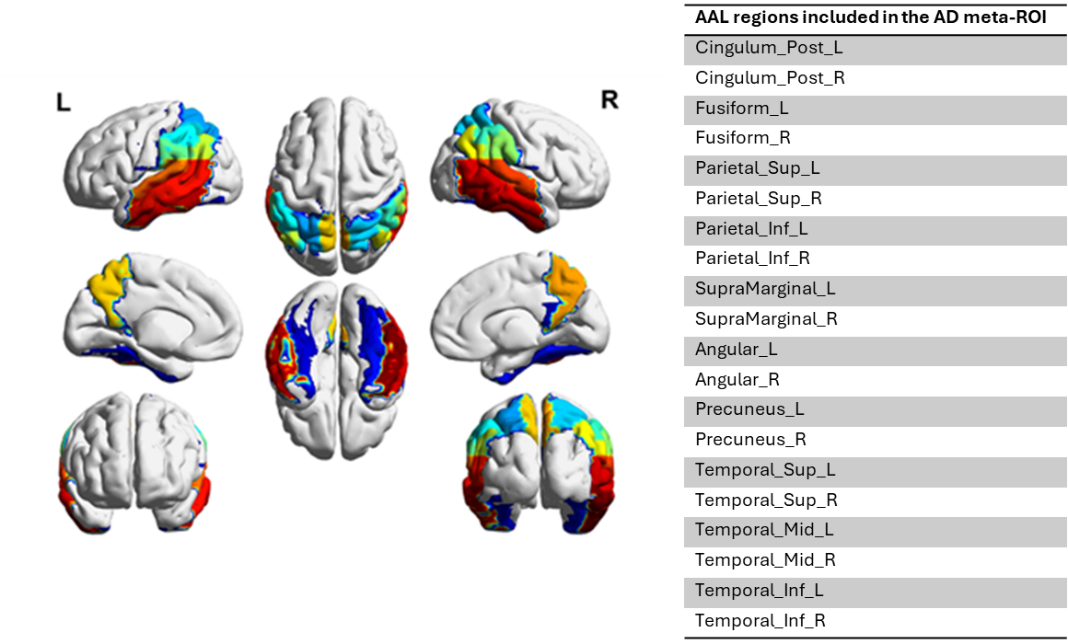


**References**

1. Guedj E, Varrone A, Boellaard R, et al. EANM procedure guidelines for brain PET imaging using [18F]FDG, version 3. *Eur J Nucl Med Mol Imaging*. 2021.

2. Boellaard R, Delgado-Bolton R, Oyen WJG, et al. FDG PET/CT: EANM procedure guidelines for tumour imaging: version 2.0. *Eur J Nucl Med Mol Imaging*. 2015;42:328-354.

3. Shcherbinin S, Schwarz AJ, Joshi A, et al. Kinetics of the tau PET tracer 18F-AV-1451 (T807) in subjects with normal cognitive function, mild cognitive impairment, and Alzheimer disease. *J Nucl Med*. 2016.

4. Fleisher AS, Pontecorvo MJ, Devous MD, et al. Positron Emission Tomography Imaging with [18F]flortaucipir and Postmortem Assessment of Alzheimer Disease Neuropathologic Changes. *JAMA Neurol*. 2020.

5. Mathoux G, Boccalini C, Peretti DE, et al. A comparison of visual assessment and semi-quantification for the diagnostic and prognostic use of [18F] flortaucipir PET in a memory clinic cohort. *Eur J Nucl Med Mol Imaging*. 2024:1-12.

6. Savio A, Fünger S, Tahmasian M, et al. Resting-state networks as simultaneously measured with functional MRI and PET. *J Nucl Med*. 2017;58:1314-1317.

7. Perani D, Della Rosa PA, Cerami C, et al. Validation of an optimized SPM procedure for FDG-PET in dementia diagnosis in a clinical setting. *NeuroImage Clin*. 2014;6:445-454.
